# Supplementary material for: MetaRibo-Seq measures translation in microbiomes
Source: Nat Commun. 2020 Jun 29;11:3268. doi: 10.1038/s41467-020-17081-z (PMC7324362; doi:10.1038/s41467-020-17081-z)
Supplement: Supplementary file 10 — Supplementary Data 7 [file 41467_2020_17081_MOESM10_ESM.zip › File2/Confidence_High_Taxonomy/338422_out.krona.html]

Javascript must be enabled to view this page.

members
magnitude
magnitudeUnassigned
count
unassigned
taxon
rank

338422\_out

3

2759
3
superkingdom

4751
3
kingdom

subkingdom
3
451864

phylum
3
4890

subphylum
147538
3

3
147550
class

222544
3
subclass

639021
3
order

81093
3
family

genus
3
228882


SRS023914\_contig\_number\_contig-100\_1471.161817SRS051031\_contig\_number\_contig-100\_1456.196315SRS053356\_contig\_number\_29791
species
3
165778
